# Amyotrophic Lateral Sclerosis: A Diet Review

**DOI:** 10.3390/foods10123128

**Published:** 2021-12-17

**Authors:** Salvatore D’Antona, Martina Caramenti, Danilo Porro, Isabella Castiglioni, Claudia Cava

**Affiliations:** 1 Institute of Bioimaging and Molecular Physiology, National Research Council (IBFM-CNR), Via F.lli Cervi 93, 20054 Milan, Italy; salvatore.dantona@ibfm.cnr.it (S.D.); martina.caramenti@ibfm.cnr.it (M.C.); danilo.porro@ibfm.cnr.it (D.P.); 2 Department of Physics “G. Occhialini”, University of Milan-Bicocca, Piazza della Scienza 3, 20126 Milan, Italy; isabella.castiglioni@unimib.it

**Keywords:** amyotrophic lateral sclerosis, diet, motor neuron degeneration, microbiota, eating behaviour

## Abstract

Amyotrophic lateral sclerosis (ALS) is a fatal disease related to upper and lower motor neurons degeneration. Although the environmental and genetic causes of this disease are still unclear, some factors involved in ALS onset such as oxidative stress may be influenced by diet. A higher risk of ALS has been correlated with a high fat and glutamate intake and β-methylamino-L-alanine. On the contrary, a diet based on antioxidant and anti-inflammatory compounds, such as curcumin, creatine, coenzyme Q10, vitamin E, vitamin A, vitamin C, and phytochemicals could reduce the risk of ALS. However, data are controversial as there is a discrepancy among different studies due to a limited number of samples and the many variables that are involved. In addition, an improper diet could lead to an altered microbiota and consequently to an altered metabolism that could predispose to the ALS onset. In this review we summarized some research that involve aspects related to ALS such as the epidemiology, the diet, the eating behaviour, the microbiota, and the metabolic diseases. Further research is needed to better comprehend the role of diet and the metabolic diseases in the mechanisms leading to ALS onset and progression.

## 1. Introduction

First described in 1869 by neurologist Jean-Martin Charcot, Amyotrophic lateral sclerosis (ALS) is a neurodegenerative disease that involves motor neurons (MNs) with onset between 50 and 65 years [1,2,3,4]. ALS may occur in a sporadic form, the most common (90–95% of cases), with no known hereditary component, or in a family-form (5–10% of cases) with a hereditary component, involving altered genes such as TAR DNA Binding Protein (TARDBP or TDP43), Superoxide Dismutase 1 (SOD1), FUS RNA Binding Protein (FUS), and C9orf72-SMCR8 Complex Subunit (C9orf72) [5,6,7,8]. Mutations in SOD1 were prevalent in Scandinavia and mutations in TDP43 in Sardinia population [8]. Genetic susceptibility has been reported by association studies that revealed also several potential ALS risk genes such as Solute Carrier Organic Anion Transporter Family Member 1B1 (SLCO1B1), Thiopurine S-Methyltransferase (TPMT), and Peripheral Myelin Protein 22 (PMP22) [9]. On the other hand, there are several environmental risk factors including the exposures to specific toxins, agricultural chemicals and smoking that could have an immediate impact on the lower motor neuron synapse [10,11].

This disease is not only widely heterogenic at a genetic, clinical and neuropathological level [12,13], but it is also characterized by a non-homogeneous spread around the world, with a particular difference in onset frequency between Western countries, such as European countries (2.1 to 3.8 cases per 100,000 person-years) [14,15,16,17,18], and Eastern countries, such as South Korea (1.2 cases per 100,000 person-years) [19] and China (0.8 cases per 100.000 person-years) [20]. The difference in geographic distribution of ALS could suggest the use of genetic tests for ALS patients to better understand the genetic landscape of the disease and an effective therapy [21]. However, ALS is rare before age 50 years and incidence of ALS should be tempered by age corrections [2]. Despite the dramatic progression of the disease an early ALS diagnosis could avoid unnecessary invasive treatments [4].

Oxidative stress (OS), high levels of reactive oxygen species (ROS), and mitochondrial dysfunction have a crucial role in patients with a neurodegenerative disease [22]. Indeed, elevated levels of OS biomarkers and ROS have been identified in the central nervous system of ALS patients [23].

A correlation among these processes in ALS has been demonstrated with several life-style factors, such as diet, alcohol, tobacco, sedentary lifestyle, or exposure to toxic materials [24]. Riluzole, an anti-glutamate agent, is the only treatment approved by the US Food and Drug Administration and the European Medicine Agency (EMA) and it can increase the survival of ALS patients by a few months [25]. However, to date there is no effective cure, but the use of antioxidant compounds could be a potential therapeutic strategy as they could help regulate the crucial biological processes involved in ALS [22].

Compounds with antioxidant potential are present in our diet such as vitamins, curcumin, and Coenzyme Q10 and could be used as therapeutic strategies. However, previous studies demonstrated also possible adverse effects of high fat intake and glutamate in ALS [22]. Moreover, emerging studies suggested that diet can influence the microbiome, and may be associated to neurodegenerative diseases, and directly correlated to energy metabolism [26]. Previous studies identified an altered metabolism in ALS patients, and a study of ALS mouse models demonstrated that the metabolic modifications could be the cause of the disease. These studies suggest that a proper diet could be a strategy to enhance the metabolic changes and prevent the disease [27].

The purpose of this review is to describe the impact of diet on ALS. We summarized the mechanism of action of antioxidant compounds, and we suggested them as potential therapeutic and prevention strategy.

## 2. Discussion

### 2.1. Epidemiology

Different countries have different frequency of ALS onset. Although the world average incidence of this disorder is about 1.9 cases per 100,000 person-year [28], epidemiological studies indicate that the highest incidence of ALS occurs in Caucasian populations, while it remains low in African, Asian and Hispanic populations [29]. Indeed, literature reports that some Western states have a high ALS onset rate, like Sweden and Scotland with 3.8 cases per 100,000 person-year [14,15,16,17]. On the contrary, Eastern states like China with 0.8 cases per 100,000 person-years and South Korea with 1.2 cases per 100,000 person-years are described as places where the incidence of ALS is particularly low [19,20]. However, there are some exceptions: despite Japan being an Eastern country, literature reports a very high ALS rate on the Kii peninsula, with 2.2 cases per 100,000 person-year. On the other hand, in the Western context, Germany (Rhineland-Palatinate) has a lower ALS onset rate than the average of other European countries, with 1.8 cases per 100,000 person-years [30,31].

**Figure 1:**
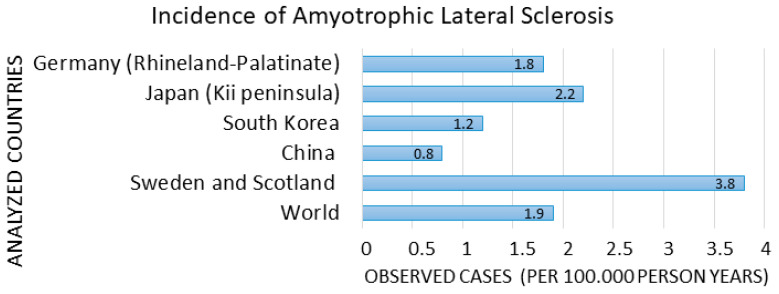
Incidence of Amyotrophic Lateral Sclerosis between Germany, Japan, South Korea, China, Sweden and Scotland and the World.

Regardless of current differences, the ALS incidence is predicted to rise in the near future around the world, mainly due to the lengthening of life expectancy and consequently the increase in the number of individuals aged 60–79 years, those most at risk of developing ALS [29]. This predicted increase is estimated also considering several risk factors based on lifestyle and environment which are rising [32]. This scenario will be particularly noticeable in the developing countries, which are expected to show a rise of 50% of the number of cases from 2015 to 2040. Instead, for the more advanced countries, an increase of 24% of the number of cases is expected from 2015 to 2040 [29]. Figure 1 summarizes the incidence of cases among the aforementioned countries.

### 2.2. Diet and Prevention

The causes of the onset of ALS are unknown. However, it has been reported that multiple aspects could influence the onset of ALS such as OS, inflammation, mitochondrial dysfunction, genetic and environmental factors [9].

Some of these factors such as OS may be influenced by diet as intake of antioxidants could decrease OS [33]. In addition, emerging evidence suggests that dietary habits in different countries could explain the difference in the incidence of ALS around the world [34,35].

For example, literature reports that the high incidence of ALS in the Kii peninsula of Japan could be due to β-methylamino-L-alanine (BMAA). BMAA, a natural neurotoxic non-protein amino acid, is produced by a symbiotic cyanobacteria in the roots of the cycad (*Cycas micronesica*) seeds that are particularly present in this area. It is hypothesized that ALS patients in this region cannot prevent BMAA accumulation [36,37,38]. The dietary source of BMAA could be seafood such as fish, mussels, crabs, and oysters [39,40,41]. High incidence of ALS has also been reported on the island of Guam whose population use potential cycad-derived products. Another possible source of BMAA contamination could be fruit bats or flying foxes as they consume cycad seeds, and they are a component of the local population’s diet [36]. Although more studies are needed, recently Martin and colleagues showed a relation between BMAA and microcystin leucine and arginine (other cyanotoxins) with the neurodegeneration, using a larval zebrafish model [42]. In addition, new-born rats exposed to BMAA were affected by motor defects, suggesting that exposure during neural development could lead to develop ALS [43]. Previous studies investigated the mechanisms of action of BMAA on the neurodegeneration: BMAA kills NADPH-diaphorase-positive motor neurons and plays a toxic role to glial cells affecting motor neuron injury [44,45].

The excessive intake of fat foods in ALS patients, and in particular saturated fat food [46], and the loss of defence mechanisms against ROS, such as the mutation of the SOD1 gene [47], are the main aspects presented in ALS patients. It is therefore reasonable to associate the large consumption of lipid foods in certain countries with a greater probability of finding cases of ALS. Thus, this could partially explain why the ALS incidence is so high in states like Sweden and Scotland [14,17], known to have a diet particularly focused on the consumption of fat food. However, the role of fat intake in ALS is debated as different studies showed contrary results [48]. Nelson et al. demonstrated that a high intake of fat is correlated with ALS onset [49]. Another study showed an opposite result: a reduced risk to develop ALS in subject with a higher intake of fat [50].

Adverse effects of glutamate have been reported in ALS. It is the main excitatory neurotransmitter in the brain, and high levels of glutamate present in mushrooms, milk and protein-rich foods can lead to elevated levels of intracellular calcium which could promote neuron death [51].

Although there are foods that seem to predispose to the onset of ALS due to the release of ROS, there are also foods and compounds that might be able to prevent the disease and slowing its course [52,53].

Curcumin, a seasoning obtained from the rhizomes of *Curcuma longa*, which belongs to the Zingiberaceae family, could have beneficial effects against neurodegeneration due to its anti-inflammatory and antioxidant properties [54,55,56,57] that have been demonstrated in experimental animal models [56,57,58]. Unfortunately, despite the positive results the clinical efficacy of curcumin is still debatable [59]. However, given the strong activity of curcumin as an antioxidant, it could have a crucial role in neuron degeneration [60]. Indeed, increased levels of reactive oxygen species (ROS) stimulate the transcription of proinflammatory genes and the release of cytokines, such as Tumor Necrosis Factor—alpha (TNF-α), interleukin 1 and 6 and chemokines that cause neuroinflammatory processes. As a result, chronicity of neuroinflammation can be considered responsible for neuron degeneration [61]. Several studies in mouse models reported that curcumin reduces OS conditions increasing the levels of antioxidants such as glutathione, and superoxide dismutase [62,63]. In particular, literature reports the overexpression and presence of mutated version of TAR-DNA-binding protein of 43 (TDP-43) in familial ALS. The consequence is its aggregation and mislocalization in the neuritis or cytoplasm [64,65,66]. Lu and colleagues analysed the potential role of curcumin as a treatment using a cellular ALS-like model generated by mutated human TDP-43. They demonstrated that dimethoxy curcumin, present in curcumin, has a protective effect on mitochondrial membrane potential, decreasing the levels of uncoupling protein 2 [67,68]. A clinical study demonstrated that 1 year of treatment with nanocurcumin and Riluzole increased the survival rate in ALS patients [69]. Curcumin shows no adverse toxicological effects in rats [70], as well as in humans [71,72,73]. Nevertheless, some patients showed episodes of diarrhoea and nausea in dose-response studies as possible side effects [74,75].

Creatine is another dietary supplement that deserves attention for its beneficial effects. It is an endogenous compound synthesized from arginine, glycine and methionine [76]. Since most of the creatine is stored in skeletal muscle, athletes are used to integrating it into their diet, in order to improve their muscle tone. Recent studies described new uses for creatine that may be useful in the prevention or in the delay of the onset of neurodegenerative diseases. In particular, Klivenyi and colleagues observed that a long-term creatine supplementation leads to better survival and improved motor coordination [77]. They measured the neuroprotective effects of creatine, studying transgenic mice with an altered version of SOD1 gene. The results showed that creatine administration protected neurons from oxidative damage [77]. No adverse side effects were reported in athletes with creatine supplementation [78,79]. However, two clinical trials completed in 2003 and 2004 tested oral creatine supplementation and provided only little notable improvements in lifespan and muscle strength in patients with ALS [80,81]. Therefore, more studies are required to understand the actual amount of the effect of creatine and for this reason, the Northeast Amyotrophic Lateral Sclerosis Consortium (NEALS) is currently analysing the long-term effects of creatine supplementation [76].

Coenzyme Q10 (CoQ10) or ubiquinone, a lipid that is produced endogenously and that is present in our diet, plays a role as cofactor of mitochondrial respiratory system. Ubiquinol, the reduced form of CoQ10, acts as antioxidant and has anti-inflammatory properties [82]. It avoids the formation of free radicals, changes of proteins, lipids, and DNA, and reduces concentrations of lipid peroxidation. In addition, in many diseases, including neurological diseases, an association between the increase of ROS and a deficiency of CoQ10 has been noted [83]. Several studies reported the beneficial effects of CoQ10 in different pathologies such as hypertension [84], fibromyalgia [85] and male infertility [86]. CoQ10 was also used in several neurodegenerative diseases such as ALS and Parkinson’s disease [87]. A sufficient quantity of CoQ10 can be obtained with a balanced diet, but a supplement may be recommended in fragile subjects. Although CoQ10 is well-tolerated, the studies are limited in pregnant women and children. CoQ10 could lead to some side effects as diarrhoea, vomiting, and rash. In addition, CoQ10 could decrease the therapeutic efficacy of several drugs such as warfarin [88].

Vitamins are involved in the development of the nervous system and could serve as prognostic factors. They may also be used in the treatment of ALS for their cellular antioxidant properties [89]. They are normally well tolerated and should not cause significant adverse effects [90,91,92]. However, their use as supplements is debated. For example, the effect of vitamin E supplementation on cognitive functions and neurological diseases is controversial. Several studies identified no effect in patients with cognitive deficit or Alzheimer’s disease [93,94]. Other studies found a beneficial effect as vitamin E could reduce OS markers after 3 months of vitamin E supplementation with Riluzole in ALS patients. However, vitamin E did not influence the survival in patients [95,96]. Recent studies suggested that vitamin E also possesses regulatory functions, including signal transduction, the inhibition of protein kinase C activity, the inflammation responses, and the gene expression regulation [96]. A high intake of vitamin E (in association with polyunsaturated fatty acid such as omega 3, present in fish and algae oil) is correlated with a 50–60% decreased risk of developing ALS [33]. Although vitamin E supplementation could have a protective role in neurodegenerative diseases its efficacy remains to be shown.

Another vitamin with a potential role in ALS is vitamin C. Limited studies have been conducted and with a small number of samples. For example, Padayatty et al. demonstrated that the supplementation of vitamin C in animal models before ALS does not influence its onset, but it reduces the progression of paralysis induced by the disease [97].

Low levels of vitamin A have been reported in neurodegenerative diseases such as Parkinson’s and Alzheimer’s disease [98,99]. However, there are conflicting results on the role of vitamin A in ALS patients [100]. Fitzgerald et al. reported that a high intake of vitamin A contained in carotenoids was correlated with a lower risk of ALS onset [101]. Other studies found no significant association between vitamin A and ALS [102].

The low incidence of neurodegenerative diseases in China could be due to the wide consumption of fruit and vegetables, which is linked with high presence of phytochemicals with high ROS scavenging [103].

Previous studies demonstrated that bioactive compounds derived by the plant, known as phytochemicals, have a neuroprotective role in neurodegenerative diseases. Indeed, a growing number of studies underlies their antioxidant properties [104,105]. Phytochemicals are present in vegetables, cereals and fruits and they are often described in literature as “nutraceutical” [106].

Phytochemicals include a wide range of chemical compounds, such as carotenoids, phenolic compounds, and terpenoids [104].

Carotenoids are a wide range of plant pigments present in many fruits, giving the typical red, yellow, and orange colour. Their targets are the peroxyl radicals [107]. They are also precursors of Vitamin A, another antioxidant. Literature reports synergistic effects in scavenging reactive nitrogen species between β-carotene and vitamins E and C [108]. Previous studies demonstrated that the intake of carotenoids is inversely correlated with ALS risk [101].

Polyphenols a class of compounds is consisting of a wide range of molecules. It is characterized by the presence of at least one phenol ring, important for the antioxidant and antitumor activity, with hydroxyl, methyl, or acetyl groups replacing the hydrogen [109,110,111]. It seems that the scavenger activity is related by the free number of hydroxyls and conjugation of side chains to aromatic rings [111]. Several studies performed in ALS animal models demonstrated that polyphenols have a neuroprotective role [112]. Flavonoids are the major of phenolic compounds. They belong to a large group of plant pigments whose chemical structure is derived from flavone. They are composed of the following subclasses: anthocyanidins, flavanones, flavan-3-ols, flavones, flavonols and isoflavones. It is deemed that the positive activities of phenolic compounds may be related to apoptosis, antioxidant, prooxidant characteristics and scavenging of radicals [113]. The flavonoids play a role in neuroinflammation silencing the microglial activation and interacting with neuronal receptors [114]. Human neuronal SH-SY5Y neuronal cells, a model of neurodegenerative disease, were treated with several flavonoids, namely quercitrin, isoquercitrin, and afzelin. The treatment showed beneficial effects downregulating the expression of cyclooxygenase-2, and apoptotic pathway [115]. Resveratrol (3,5,4′-trihydroxystilbene) a polyphenol presents in grapes, berries, and peanuts could be an interesting neuroprotective compound [116]. It regulates Sirtuin 1 (SIRT1), the major member of sirtuin deacetylates proteins, modulating gene expression through epigenetic gene silencing. A study demonstrated that Resveratrol increases the SIRT1 expression in the cortex and hippocampus reducing the cognitive impairment [117].

Terpenoids are a very large family of plant secondary metabolites [118]. In vitro, it has been showed that diterpenes, monoterpenes and sesquiterpenes extracted from aromatic plants have notable antioxidant activity suggesting them as compounds against neurodegeneration [119].

Not all natural compounds that showed significant health benefits have also a neuroprotective role in neurological diseases. For example, omega-3 supplementation in mouse models of ALS reported an increased cellular damage that could increase disease progression [120]. Similar results were obtained in a more recent study in a murine model of familial ALS [121]. However, combination of omega-3 and vitamin E could reduce ALS risks [33].

Generally, it seems that a good anti-ALS food or compound has to possess at least one of these qualities: anti-inflammatory or antioxidant property, since the OS and the inflammation play an important role in the neuron degeneration [122].

#### Food-Related Exposure to Toxicants

While ALS pathogenesis has not been fully elucidated yet, it is known that in the sporadic origin genetic factors and environment interact with each other, facilitating disease onset in genetically predisposed individuals [123]. Among environmental factors, studies have identified neurotoxic chemicals such as heavy metals and pesticides as possible risk factors for the development of ALS, but evidence is limited [123,124,125].

While exposure to toxicants may occur in a wide range of occupational settings and in the general population through different means such as air pollution [126] or smoking [127,128] among others, factors not related to diet are beyond the scope of this review. Different mechanisms may lead to food-related toxicants exposure, such as bioaccumulation, the accumulation of toxic substances in the tissues of an organism [129], and biomagnification, which indicates the increased concentration of toxicants based on the position in the food chain [129], but also contamination by food containers or pesticides and dietary supplements [130,131,132].

In particular, exposure to metals has been suggested to be a possible risk factor for ALS, but results are not conclusive. Studies show that cadmium and lead may be associated with an increased risk of developing ALS and zinc with a decreased risk based on pre-disease metal levels in blood, with lead having the strongest a priori connection [133,134,135].

Also, mercury has been suspected to be part of ALS pathogenesis [136], but results are inconclusive, especially regarding mercury exposure from diet, and in particular seafood consumption [137]. Mercury is produced by several industries and stores in aquatic predatory organisms such as shark, swordfish, mackerel and tuna [138]. Mercury can generate oxygen free radicals, promote excitotoxicity, and decrease DNA, RNA and protein synthesis, [139], all processes that have been associated with ALS [140]. However, several studies reported that people with and without ALS are exposed to the same amount of mercury. The difference could be that ALS patients are more susceptible to mercury due to genetic/epigenetic predispositions [141].

The discrepancies among different studies could be due to the fact that single metal analyses may not be able to fully evaluate the relevance for health risks [142], suggesting the likelihood of the interaction of toxicants exposure with additive or synergistic effects [142].

### 2.3. Eating Behaviour

As mentioned above, one of the principal factors in the ALS onset seems to be a lipidic-focused diet, playing a crucial role in neurodegeneration due to the high release of ROS [61]. Recent studies showed that presymptomatic ALS patients might increase total daily energy consumption compared to healthy individuals [48] and that high-caloric food supplements with high fat levels could stabilize weight loss in patients with advanced ALS [143]. This could be explained by metabolic alterations as reported by a study in presymptomatic mice [144].

**Figure 2:**
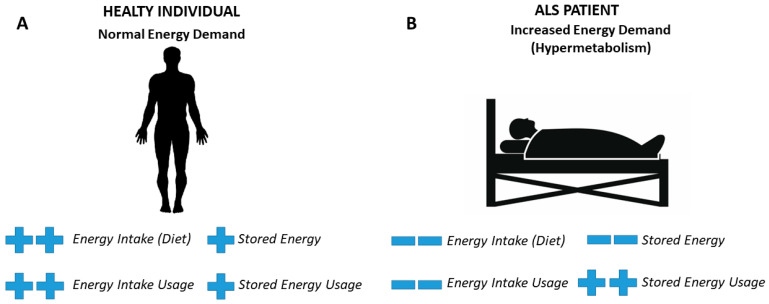
Metabolic differences between a healthy individual and a patient affected by Amyotrophic Lateral Sclerosis (ALS). (**A**) In healthy individuals, during periods of normal energy demand, energy intake is used to meet energy demands, but when there is excess energy, it is stored in the fatty tissue and in the liver. Failure to maintain energy supply leads to a negative energy balance and in this case, the energy reserves in the adipose tissue and liver are used to meet the energy needs. (**B**) Hypermetabolism, i.e., an increase in energy demand, occurs in ALS. Indeed, in ALS a decreased energy intake results in decreased storage of energy in the adipose tissue and liver, and an increased dependence on the use of stored energy. Therefore, the decrease in body mass index in ALS is a consequence of negative energy balance and hypermetabolism [150].

The insufficient food intake and weight loss due to dysphagia and loss of appetite, typically present in ALS patients, may reflect a condition of hypermetabolism and increased catabolic demand [145] (Figure 2). This could lead to an increase of the caloric intake as a compensatory measure through the intake of fatty foods in ALS patients [46].

Changes in eating behaviour have also been observed in subjects affected by front temporal disease (FTD), which shares a significant overlap at genetic, pathological and behavioural levels with ALS [146]. This preference for lipidic foods is further marked in subjects in both diseases [46]. In a recent study, Ahmed and colleagues measured the concentrations of some peptides responsible for appetite in patients with ALS and FTD and demonstrated that, compared to the control group, all the subjects exhibited elevated levels of insulin and leptin, responsible for the feeling of satiety. In contrast, peripheral neuropeptide Y (NPY) levels, not only correlated with eating behaviour, but also with the disease duration, and were significantly increased in ALS patients and decreased in FTD patients [146].

The hypothalamus seems to be another aspect to take into account for the occurrence of the anomalous dietary behaviours in FTD and ALS patients and that can be correlated to these peptides. Indeed, the hypothalamus plays a central role in any change in eating-peptides and metabolic status [146]. Specifically, previous studies showed a correlation between hypothalamic atrophy, represented by a reduced volume, and FTD [147,148] and ALS [149]. Particularly, Gorges and colleagues noticed that the volume loss occurred not only in patients affected by these disorders, but also in presymptomatic ALS mutation carriers, even before the onset of symptoms related to motor neuron degeneration [149]. Therefore, the development of anomalous dietary behaviours could be an important marker for early recognition of the occurrence of ALS [46], despite more studies are needed in order to understand whether these changes in diet and consequently in metabolism represent a pathogenic factor or an adaptive mechanism during the ALS occurrence.

### 2.4. Microbiota and Microbioma

During the last few years, the scientific community has reported the potential role of gut microbiota in metabolic and immunity control since it has a symbiotic relationship with the host organism [151]. The gut microbiota is defined as the bacterial population only present at the gastrointestinal (GI) level.

Small molecular metabolites produced by gut bacteria and circulating into the blood regularly mediate the communication between the human brain and the gut microbiome influencing many brain processes, such as myelination, and neurogenesis [152].

A proper diet generates a good gut microbial community and brain health. On the contrary, the normal brain processes can be altered by a diet characterized by a high intake of sugar and fat [153].

The gut microbiota has been identified as a risk factor in the onset of different neurological disorders, including ALS [154,155,156].

An important study that demonstrated a link between ALS and gut microbiome, i.e., the genes expressed by the microbiota, was conducted by Wu and colleagues [157]. They identified damage to intestinal barrier function and decreased levels of butyrate-producing bacteria in the SOD1 mouse model. Butyrate could play a role in neurological disorders since it regulates energy metabolism and immune functions [157].

As demonstrated by an experimental autoimmune encephalomyelitis model, some tryptophan metabolites involved in the pathway of inhibition of neuroinflammation and neurodegeneration and produced by the microbiota can regulate microglia and astrocytes present in the central nervous system [158]. Other evidence supporting the role of the microbiota was reported in a study on the G93A-SOD1 transgenic mice models of ALS [159]. The researchers showed three important aspects: the first was the role of impaired intestinal epithelium and tight junction in ALS progression [159]. The second aspect was that replenishing the mice with probiotics and the relevant metabolites helped ameliorate the motor ability [160,161]. The third aspect was that the gut microbiota in mice was altered prior the development of motor neuron degeneration. This means that dysbacteriosis could be one of the possible mechanisms influencing ALS onset [161]. In addition, Blacher et al. demonstrated that the supplementation of commensal bacteria *Akkermansia muciniphila* has a neuroprotective role against ALS pathogenesis, whereas *Ruminococcus torques* and *Parabacteroides distasonis* worsen the disease [161].

### 2.5. Metabolic Disease and ALS

The literature reported that metabolism plays a central role in ALS onset and its course. In line with this scenario, it is interesting to investigate if there are specific metabolic diseases that could share some genetic risk loci with this disorder.

Metabolic defects have often identified in ALS patients. However, there are controversial opinions of the scientific community: it is not clear if the metabolic alterations are a consequence of ALS or if they could play a role in the disease onset [25,162].

Mendelian randomization analyses indicated that there is a causative association between obesity-related factors and ALS. For example, ALS risk is positively associated with low-density lipoprotein cholesterol level (LDL-C) [163].

Despite the loss of weight in ALS patients, a genome-wide association study (GWAS) conducted by Li and colleagues demonstrated that there is some evidence of a correlation between ALS and 5 obesity related traits: body mass index (BMI), body fat percentage (BFP), high-density lipoprotein cholesterol (HDL-C), low-density lipoprotein cholesterol (LDL-C), and type 2 diabetes (T2D). This study highlighted 5 risk genes: Sec1 Family Domain Containing 1 (SCFD1), Ataxin 3 (ATXN3), Gametogenetin Binding Protein 2 (GGNBP2), C9orf72 and DENN Domain Containing 6B (DENND6B) [163]. SCFD1 and ATXN3 are involved in the regulation of protein processing, transport and metabolism [164]. The abnormal protein metabolism has been observed in both obesity and ALS [165]. Some studies found that GGNBP2 is associated with BMI and waist-hip ratio [166]. GGNBP2 is also a tumour suppressor involved in several kinds of cancers [167]. C9orf72, whose expansion of repetitions is a common cause of ALS, was identified as a shared risk gene for ALS and HDL-C/LDL-C [167]. A recent study showed that decreased serum levels of HDL-C were observed in subjects C9orf72 repeat expansion carriers. This observation suggests that the abnormal lipid metabolism could be associated with the pathogenic mechanism of the C9orf72 repeat expansion mutation [167]. DENND6B plays a role in vesicle-mediated transport and RAB GEFs exchange GTP for GDP on RABs, that are involved in ALS [168].

The role of BMI remains controversial. Indeed, a study conducted by Nakken and colleagues showed that high early-aged levels of BMI are associated with low ALS risk several decades later [169]. Moreover, a study conducted by Goutman and colleagues showed that higher premorbid BMI is associated with slower ALS progression [170]. A high BMI at diagnosis is also associated with a better survival suggesting it as a marker of disease severity [170].

The good survival observed in ALS patients with a high BMI could also indicate that an hyperalimentation resulting in an increasing BMI could improve the prognosis of ALS patients (clinical trial NCT00983983).

On the other hand, it is interesting to note that the obesity rate is particularly high in Sweden and low in China and South Korea (https://data.worldobesity.org/rankings/, access 15 June 2021), with high levels of onset of ALS in Sweden, as opposed to the onset in China and South Korea. Another interesting aspect is that the obesity rate in males is higher than in females, but there is also a greater incidence of ALS in males than in females [171]. Further studies are needed to investigate the possible relation between obesity and ALS onset and progression.

In contrast, T2D could increase Alzheimer’s and Parkinson’s diseases onset, but could have a protective effect against ALS [172]. An interesting study was conducted by Tsai and colleagues [173]. They showed that a late onset of T2D may have a negative association with ALS, especially when combined with hypertension, but an onset of T2D before the 55 years may have a positive association with ALS, especially when combined with hyperlipidaemia [174].

In a recent study Chen et al. found an inverse correlation and 8 shared pleiotropic genes between T2D and ALS [175]. Although more studies are needed to clarify the underlying mechanisms that can justify this effect, previous research reported the same effects in the Danish and Italian populations [176,177]. However, the inverse correlation between T2D and ALS could be associated with the ethnic background as in the Asian population diabetes is a risk factor of ALS [176,177].

The molecular mechanism behind the relation between T2D and ALS is not completely clear [161]. A possible explanation could be due to the known involvement of TAR DNA-binding protein 43 kDa (TDP-43) in T2D and ALS [177,178]. Indeed, TDP-43 is involved in the formation of cytoplasmatic aggregation in motor neurons and can also regulate glucose transport by TBC1 [179,180].

Moreover, several studies suggested a potential protective effect of anti-diabetic drugs on ALS onset. Specifically, they studied the role of pioglitazone, a common drug used in the treatment of diabetes, in ALS patients. The anti-oxidant and anti-inflammatory properties of pioglitazone make it a potential candidate for ALS treatment [181,182].

However, the causes of metabolic defects are unclear, and few studies investigated their role in patients before the onset of ALS.

## 3. Conclusions

Although the central role of food in daily life is known, more attention has to be paid to this aspect, especially in the medical field. Indeed, a better education and study of diet can not only favour the recovery from some diseases, but it can also help to prevent them and consequently improve the quality of life. The environmental and genetic causes that lead to the onset of ALS are numerous and still to be clarified, but we believe that a study of the dietary patterns of Eastern countries with low ALS rates, such as China or South Korea, may be the key to a deeper understanding of mechanisms to prevent and treat this disease. Furthermore, if we fully understood why certain foods can harmful or beneficial in ALS, especially combined with other risk factors, we would consequently have a greater understanding of some aspects linked to this still rather unknown disease. In fact, the mechanisms of development of ALS are complex and diagnosis is not easy since the disease may present with a wide variety of clinical symptoms, which could be very subtle and easily overlooked at the onset, leading to a delayed diagnosis that could limit the crucial early control of the symptoms. A better understanding of the interaction of the different risk factors in the onset and progression of ALS could be crucial especially when considering easily modifiable aspects of daily life such as diet. However, we noted that the data regarding the potential benefits of diet are conflicting in the different studies. Our review highlights the gaps in the literature: (1) most of the studies are performed with small sample sizes, (2) there is not a standardized approach to evaluate the impact of the diet on a disease, (3) many variables could influence the results obtained from different studies such as age of patients, environment conditions or genetic susceptibility, but also many other aspects linked to lifestyle and to the interaction of the different variables.

## Data Availability

The data that support the findings of this study are openly available in https://pubmed.ncbi.nlm.nih.gov/ (accessed on 12 December 2021). For more information, refer to the articles DOIs in the bibliography.
